# Chronic Periodontitis and Immunity, Towards the Implementation of a Personalized Medicine: A Translational Research on Gene Single Nucleotide Polymorphisms (SNPs) Linked to Chronic Oral Dysbiosis in 96 Caucasian Patients

**DOI:** 10.3390/biomedicines8050115

**Published:** 2020-05-09

**Authors:** Francesco Inchingolo, Francesco Saverio Martelli, Ciro Gargiulo Isacco, Elisa Borsani, Stefania Cantore, Fabiana Corcioli, Anna Boddi, Kieu C.D. Nguyễn, Danila De Vito, Sergey K. Aityan, Van Hung Pham, Gianna Dipalma, Andrea Ballini

**Affiliations:** 1 Department Interdisciplinary of Medicine, University of Bari “Aldo Moro”, 70121 Bari, Italy; francesco.inchingolo@uniba.it (F.I.); francesco.martelli@ednmail.it (F.S.M.); drciroisacco@gmail.com (C.G.I.); stefaniacantore@pec.omceo.bari.it (S.C.); kieugmv@gmail.com (K.C.D.N.); giannadipalma@tiscali.it (G.D.); 2 International Microdentistry Institute (IMI), 50139 Florence, Italy; 3 Department of Basic Medical Sciences, Neurosciences and Sense Organs, University of Bari “Aldo Moro”, 70121 Bari, Italy; danila.devito@uniba.it; 4 Division of Anatomy and Physiopathology, Department of Clinical and Experimental Sciences, University of Brescia, 25123 Brescia, Italy; 5 Biomolecular Diagnostic S.r.l., 50139 Florence, Italy; fabiana.corcioli@biomoleculardiagnostic.com (F.C.); anna.boddi@bdmail.it (A.B.); 6 Human Stem Cell’s (HSC), Ho Chi Minh City 70000, Vietnam; 7 Department of Multidisciplinary Research Center, Lincoln University, Oakland, CA 94612, USA; aityan@lincolnuca.edu; 8 Pham Chau Trinh University of Medicine, Da Nang City 50000, Vietnam; phhvan.nkbiotek@gmail.com; 9 Department of Biosciences, Biotechnologies and Biopharmaceutics, Campus Universitario “Ernesto Quagliariello”, University of Bari “Aldo Moro”, 70125 Bari, Italy; andrea.ballini@uniba.it; 10 Department of Precision Medicine, University of Campania“Luigi Vanvitelli”, 80138 Naples, Italy

**Keywords:** chronic periodontitis, oral dysbiosis, IL-10, clinical biochemistry and clinical molecular biology, single nucleotide polymorphisms (SNPs), translational research

## Abstract

Chronic periodontitis (CP) is a complex pathology with a significant impact worldwide causing bone loss. Oral dysbiosis is a highly inflammatory condition associated to a long-term insulting infection and represents an underestimated CP key factor associated with an imbalance of pro-inflammatory and anti-inflammatory gene responses. The presence of a single nucleotide polymorphisms (SNPs) in the promoter region of interleukin 10 (IL-10) gene −1082, −819, and −592 was a possible determinant cause. This translational research aimed to provide outcomes on the role of IL-10 gene expression in bone loss diseases in patients affected by CP. Caucasian patients (*n* = 96) affected by CP were recruited from the Italian population. The subgingival samples were collected using the Bacterial Periodontal Assessment by Biomolecular Diagnostic^®^ and the characterization of a set of 15 bacterial DNA responsible of periodontitis was performed by real-time multiplex PCR. In addition, two viruses, Epstein–Barr Virus (EBV) and Herpes Simplex Virus 1 (HSV-1), and a pathogenic fungi (*Candida albicans*) were included as a part of our panel. Our results confirmed an existing association between IL-10 gene polymorphisms and polymorphism of tumor necrosis factor alpha (TNFα), interleukin 1α-β-RN (IL-1α-β-RN), collagen type-l alpha (COLIA1), and vitamin D receptor (VDRs) genes in CP. Further studies are needed to improve diagnosis and endorse more effective therapeutic procedures for periodontal disease.

## 1. Introduction

Chronic periodontitis (CP) is a multi-factorial disorder that has showed to have a significant impact worldwide, according to recent outcomes places within the first six most prevalent chronic diseases worldwide, with more than 750 million people affected [1]. CP disease is associated with gum infection, gum bleeding, and eventually tooth and bone loss. From a broader clinical point of view, CP might be considered as part of a more complicated systemic disease often co-existent with obesity and metabolic syndrome (MetS) [2].

Different lines of evidence confirmed the primary role of MetS in increased periodontal inflammation due to the high presence of lipopolysaccharide (LPS) that immediately triggers an immune response. The experimental use of fat rats (a model of MetS) clearly confirmed the elusive high level of pro-inflammatory responses that evolved in individuals affected by both MetS and CP. The study clearly assessed the high presence of pro-inflammatory cytokines, such as the tumor necrosis factor-α (TNF-α), the granulocyte/macrophage colony-stimulating factor (GM-CSF) and the IL-6 released by the adipose tissue [2,3,4].

As a multifactorial infection, CP, if untreated, may eventually cause the insurgence of more systemic diseases. In this scenario, the balance between pro- and anti-inflammatory responses plays a key role and are under the control of local bacteria [3]. The increase of aggressive bacteria generate dysbiosis, a phenomenon that is often underestimated by the healthcare providers. Dysbiosis generates the inflammatory environment and once the inflammatory patterns are well established the entire immune/neuro/endocrine compartments progressively decline generating a cascade of deteriorating events [2,5]. The hushed chronic inflammatory condition derived from niche dysbiosis, such as oral dysbiosis, leads to the high permeability of the mucosa wall, otherwise known as the “Leaky mucosa”, a disorder that allows the free passage of intestinal/oral pathogens into the system. This event exponentially increases the magnitude of inflammation consequent to a further uncontrolled systemic auto-immune response [4,5]. 

The typical transcriptional regulator pathways of chronic inflammatory responses are fully triggered and are consequent of a relentless state of inflammation under the charge of tyrosine kinase receptors (TKRs) and the hydrolase enzymes that bind to the nucleotide guanosine triphosphate (GTPase). Furthermore, in this chronic dysbiotic environment, there is a complete overpowering of pro-inflammatory responses controlled by the gene in charge of pro-inflammatory cytokines and interleukins versus immune-modulatory gene expression. Therefore, it is highly common to see in dysbiotic patients a high level of the tumor necrosis factor (TNF) super-family such as the nuclear factor kappa B (NF-kB), the receptor activator of the nuclear factor kappa-Β ligand (Rankl), interferons (IFN), and the group of interleukins 2-4-6 (IL 2-4-6) with low levels of anti-inflammatory interleukins, like 10 (IL-10) [6,7,8,9].

Single nucleotide polymorphisms (SNPs), especially those belong to the interleukin IL-10 gene, are attracting great consideration especially when encountered in diseases consequent to oral dysbiosis as a pathological substrate of CP [10,11,12].

In periodontal diseases, the host response starts with anaerobes and Gram-positive agents. In the long-term, this phase is concluded with the colonization of Gram-negative members [1]. Generally, oral dysbiosis presents a quite wide range of periodontal pathogens that accumulate within hard tissues, tooth structures, and soft tissues [3]. The bacteria may include different strains, such as *Aggregatibacter actinomycetemcomitans*, *Porphyromonas gingivalis*, *Porphyromonas endodontalis*, *Tannerella forsythia*, *Treponema denticola*, *Prevotella intermedia*, *Peptostreptococcus micros*, *Selenomonas* spp., *Synergistetes*, *Fusobacterium nucleatum*, *Parvimonas micra*, *Campylobacter rectus*, and *Eubacterium timidum* [1]. More recent outcomes highlighted the high pathogenicity of the *Filifactor alocis* as a major agent involved in the poor course of the disease. In addition, the synergistic effect of *P. gingivalis* and *F. alocis* that significantly augmented the overall grade of infection was seen [13,14].

Nevertheless, the exact way they interface with the immune response still remains a matter of debate; definitely their bio-products, metabolites, peptides, proteins, and toxins trigger the reaction of genes involved in immune responses, such as T cells, B cells, interleukins, and cytokines. The understanding of this mechanism could open up innovative solutions in clinical therapy and health controls [15].

Thus, the proposed mechanisms may follow the direct influence of the NF-kB overexpression or be the result of the diminution of important commensal strains, such as the *Lactobacillus*, due to the dramaticly high level of aggressive pathogens typical of a highly contaminated dysbiotic environment [16].

Of note, *P. gingivalis* showed the capacity of inhibiting leukocyte recruitment in two relatively simple ways: first, by invading epithelial cells by secreting an NF-kB p65 inhibitor, known as serine phosphatase (SerB), and, secondly, by acting on endothelial cells in concert with other bacteria, this mechanism inhibits the up-regulation of E-selectin which consequently blocks the leukocyte adhesion and transmigration cascade [17,18]. It is crucial to understand that pathogens are also extremely competitive and many of them can take advantage on host immune surveillance, the majority is also able to communicate with the central nervous system for their own better survival condition. It is not rare to see this highly competitive match, for example, between *Streptococcus cristatus* and *P. gingivalis*, where the former showed to be capable to de-activate its opponent by blocking its production of fimbrial adhesin (FimA) [19,20,21].

In this light, the aim of the present translational research was to provide outcomes on gene polymorphisms (SNPs) assessed on 96 CP patients. Patient samples were collected for genetic analysis to evaluate SNPs on genes regulating IL-10 (−1082 G > A, −819 C > T, −592 C > A), TNFα (tumor necrosis factor alpha-308 G > A), IL-1α (−889), IL-1β (+3954), IL-1RN (+2018), VDRs (vitamin D receptors): Apal (+64,978 G > T) and Taql (−1056 T > C), Bsml (+63,980 G > A), Fokl (+30,920 T > C), and COLIA1 (collagen type-lα) (2046 G > T) (Table 1).

Moreover, a set of bacteria known to be involved in CP was also selected and screened for in each patient.

## 2. Materials and Methods

### 2.1. Subject Enrollment and Inclusion/Exclusion Criteria

The study was conducted in compliance with recognized international standards and the principles of the Declaration of Helsinki. In the current study, 96 patients diagnosed with CP were included: males (*n* = 43) and females (*n* = 53); smokers (*n* = 23), former smokers (*n* = 19) and non-smokers (*n* = 54). The mean age of participants was 50 ± 2 for men and 41 ± 5 for women. All subjects were in good general health conditions. To identify suitable participants, patient referral records, including a full-mouth series of peri-apical radiographs, were screened. Complete examinations of soft and hard oral tissues were performed on all patients. Exclusion criteria from the study were: diseases of the oral hard or soft tissues, except caries and periodontal disease; presence/use of orthodontic appliances; need of pre-medication for dental treatment; use of antibiotics for the preceding six-month period; pregnancy or lactation; inability or unwillingness to sign informed consent; history of diabetes; HIV positive; immunosuppressive chemotherapy; and history of any disease known to severely compromise immune function [18].

All patients were interviewed and smoking habits were recorded. They were subjected to a clinical examination of the periodontal tissues. All data from clinical and radiographic evaluation were collected into a dedicated periodontal folder. The periodontal probe (PCP15 -HuFriedy, Chicago, IL, USA) was inserted parallel to the vertical axis of the tooth and “walked” circumferentially clockwise around each surface of the tooth, to detect the area of deepest penetration (inclusion criteria: bleeding on probing and probing depth exceeding 3 mm) [18]. Our study recruited only Italian Caucasian subjects, and none of them had a history of diabetes mellitus or current manifestation of systemic diseases, which correlates with destructive periodontal disease.

### 2.2. Collection of Subgingival Samples and Characterization of Bacterial DNA Responsible of Periodontitis

In the present study, a special kit (BPA-Bacterial Periodontal Assessment BiomolecularDiagnostic^®^) was used; it was explicitly studied for the detection and the diagnosis of oral dysbiosis by the highly selective characterization of differences in the bacterial communities responsible of periodontitis and peri-implantitis.

Sampling was performed by applying the procedures described in the BPA kit after drying the area and removing the supragingival plaque. Samples from the subgingival plaque were collected with sterile paper tips inserted deeply into the periodontal pockets for one minute (at least one pocket was chosen for each quadrant) and then stored at 4 °C in a sterile tube until processing.

The bacteria analyzed were divided into three groups following the outlines of Socransky et al. [22] (Table 2):

### 2.3. RT-PCR for Bacteria, Viruses, and Fungi

After automated DNA extraction, the extracted samples were subjected to amplification and quantification through Real Time Multiplex PCR (RT-PCR) using primers and probes marked with three different fluorophores (FAM, VIC, and ROX) specific for each target.

The Multiplex technique allowed the analysis of three bacteria simultaneously. Analysis and interpretation of the results were performed according to the manufacturer’s instructions. 

For each sample of each bacterium analyzed, a value expressed in logarithm was acquired.

DNA extraction was performed using the QIACUBE HT^®^ instrument (QIAGEN^®^ GMBH, Hilden, Germany cat no./ID: 9001793). Approximately 40 ng of target DNA was used for the detection of each individual bacterium with the real-time PCR method, to assess the amount of double-stranded DNA present after each synthesis cycle in real time. In the reaction mixtures, SYBR Green, a fluorescent intercalating compound of DNA, was present. These mixtures together with specific primer pairs for each target, were seeded in each single tube with the automatic dispenser QiAgility (QIAGEN^®^, GMBH, Hilden, Germany cat no./ID: 9001532). For amplification and DNA detection, a real-time PCR (Rotor-Gene Q, QIAGEN^®^, GMBH, Hilden, Germany) was performed with species-specific primers (Table 3) for the search of the following pathogens traditionally associated with periodontal/peri-implant disease: *P. gingivalis*, *T. denticola*, *T. forsythia*, *P. micros*, *F. nucleatum* ssp., *P. intermedia*, *C. rectus*, *E. corrodens*, and *A. actinomycetemcomitans*.

In addition, two viruses, Epstein–Barr Virus (EBV) and Herpes Simplex Virus 1 (HSV-1), and a pathogenic fungi (Candida Albicans), were included in the panel (Table 3).

The reading of the two virus and candida results was carried out following the analysis of the dissociation curve (melting curve).

The Melting temperature of EBV and *Candida* was 82/84 °C, respectively, while that of HSV1 was 86 °C.

### 2.4. Analysis of Total Bacteremia Procedure Performed by RT-PCR on V3–V4 in the 16S Ribosomal RNA (rRNA)

Subsequently, the DNA of the samples subjected to the aforementioned microbiological analysis was amplified for the V3 and V4 regions of 550 bp (known sequences able to identify the bacterial species present in the sample) of the bacterial 16S rRNA gene using the RT-PCR for the following primer:

5′ TCGTCGGCAGCGTCAGATGTGTATAAGAGACAGCCTACGGGGGCGCAG 3′

3′ GTCTCGTGGGCTCGGAGATGTGTATAAGAGACAGGACTACGGGTATCTAATCC 5′

The bioinformatic analysis of the data obtained from the sequencing was also performed to identify and define the bacterial species that characterize the periodontal and peri-implant disease and to obtain a pattern of microbial species that identify and characterize the group of false negatives (Table 4).

The thermal profile used in the PCR reaction to detect the DNA of these pathogens has been performed as follows:

1 cycle × 5 min 95 °C, followed by 40 cycles ×

**Sanger sequencing**

To confirm the presence of every single analyzed bacterium, it was performed the sequencing analysis of "Sanger", using the following protocol for samples:

1 µL di Thermo Scientific FastAP Thermosensitive Alkaline Phosphatase

0.5 µL Exonuclease I (Exo I)

**Thermal profile**

37 °C × 15 min

85 °C × 15 min

4 °C × 10 min

**Marking for the sequencing reaction**:

11.5 µL H_2_O

3.5 µL Buffer 2×

1 µL BigDye^®^ Terminator v1.1 Cycle Sequencing Kit

1 µL primer 3.2 μM (micromol of concentration)

3 µL DNA purified

1 cycle × 5 min at 95 °C, for 30 cycles × $\{\begin{array}{l} \text{30 s at 95 °C}\\ \text{10 s at 50 °C}\\ \text{4 min at 60 °C} \end{array}$

### 2.5. Genotype Frequencies and Statistical Analysis

Genotype frequencies were compared between the whole set of chosen genes and the set of bacteria by chi-square analysis, followed, in the case of a significant result, by multiple comparisons. We tested the following variants according to the literature: IL-10, TNFα (tumor necrosis factor alpha −308 G > A), IL-1α (−889); IL-1β (+3954); IL-1RN (+2018), VDRs (vitamin D receptors) Apal (+64,978 G > T), Taql (−1056 T > C), Bsml (+63,980 G > A), Fokl (+30,920 T > C), and COLIA1 (collagen type-l α) (2046 G > T), *Candida albicans*, Epstein–Barr virus (EBV), and herpes simplex virus 1 (HSV-1). The genotype frequencies in patients were tested for Hardy–Weinberg equilibrium.

A chi-square test was used to determine differences in the frequencies all different genotypes between CP patients and clinical subtypes set of bacteria. The odds ratio (OR) and 95% confidence interval (95% CI) were calculated for disease susceptibility and clinical subtypes in relation to the studied IL-10 (SNPs) gene polymorphisms. Student’s t-test and analysis of variance were used to compare numeric variables within groups, depending on the distribution of the data. A *p* value < 0.05 was considered as statistically significant.

## 3. Results

### 3.1. IL-10 Correlations with VDRs, COLIA1, IL-1α (−889), IL-1β (+3954), IL-1RN (+2018), and TNF-α

There was no significant difference in age and sex distributions in the groups (*p* > 0.05).

The IL-10 genotype frequencies of all investigated groups showed disequilibrium according to Hardy–Weinberg (*p* < 0.05). The allele frequencies of the IL-10 gene polymorphisms were in accordance with Hardy–Weinberg equilibrium (*p* > 0.05). Moreover, IL-10 (−1082 G > A, −819 C > T, −597 A > C) genotype distribution was different between groups (*p* < 0.05) (Table 5). In the CP group with severe phenotype, IL-10 ATA/GCC genotypes were associated with higher risk of either periodontitis development or bone decay in comparison with IL-10 ATA/ACC-ATA/ATA-ACC/ACC genotypes, with a statistically significant risk coefficient (OR: 7, 95%CI, 2.83–60.25, *p* < 0.05). The IL-10 ATA/GCC genotypes group, in relation with TNFα (−308 G > A), IL-1α (−889), IL-1β (+3954), IL-1RN (+2018), VDRs, Apal (+64,978 G > T), Taql (−1056 T > C), Bsml (+63,980 G > A), Fokl (+30,920 T > C), and COLIA1 (2046 G > T) gene expression, showed higher levels of SNPs (Table 5). The genotype distribution in the whole CP group was 14% for the GCC/GCC genotypes (normal IL-10 expression), 45% for the ATA/GCC genotypes, and 24% for the ATA/ACC-ATA/ATA-ACC/ACC genotypes. However, allele frequencies (*p* > 0.05) were similar in CP patients with severe, moderate, and normal phenotypes for IL-10 (GCC/GCC).

### 3.2. IL-10 Production and Bacteria Presence

Bacteria present in the first group of 34 patients (IL-10 low production) were: (I) for the ATA/ACC genotypes: *A. actinomycetemcomitans* = 3 (super aggressive strain); *T. forsythensys* = 13 (high aggressive strain); *R. dentocariosa* = 13 (medium aggressive strain); *C. hominis* = 14 (low aggressive strain); (II) for the ATA/ATA genotypes: *F. alocis* = 9 (high aggressive strain); *R. dentocariosa* = 10 (medium aggressive strain); *E. corrodens* = 9 (low aggressive); (III) for the ACC/ACC genotypes: *T. forsythensys* = 9, *F. alocis* = 9, *Synergistetes* = 9 (high aggressive strain); *R. dentocariosa =* 10 (medium aggressive strain); *C. hominis* = 11 (low aggressive).

In addition, bacteria present in the second group of 40 patients (IL-10 reduced production) were: (I) for ACC/GCC genotypes: *A. actinomycetemcomitans* = 3 (super aggressive strain); *T. forsythensys* = 26 (high aggressive strain); *R. dentocariosa* = 25 (medium aggressive strain); *C. hominis* = 25 (low aggressive); (II) for ATA/GCC genotypes: *P. gingivalis* = 9, *T. forsythensys* = 9, *T. denticola* = 9, *P. micros* = 9, *Synergistetes* = 9 (high aggressive strain); *R. dentocariosa* = 9 (medium aggressive strain); *E. corrodens* = 8 (low aggressive).

Finally, for the third group of 22 patients (IL-10 normal production) bacteria present were, for GCC/GCC genotypes, *A. actinomycetemcomitans* = 2 (super aggressive strain); *P endodontalis* = 21 (high aggressive strain); *R. dentocariosa* = 21 (medium aggressive strain); *E. corrodens* = 19 (low aggressive).

## 4. Discussion

**Figure 1:**
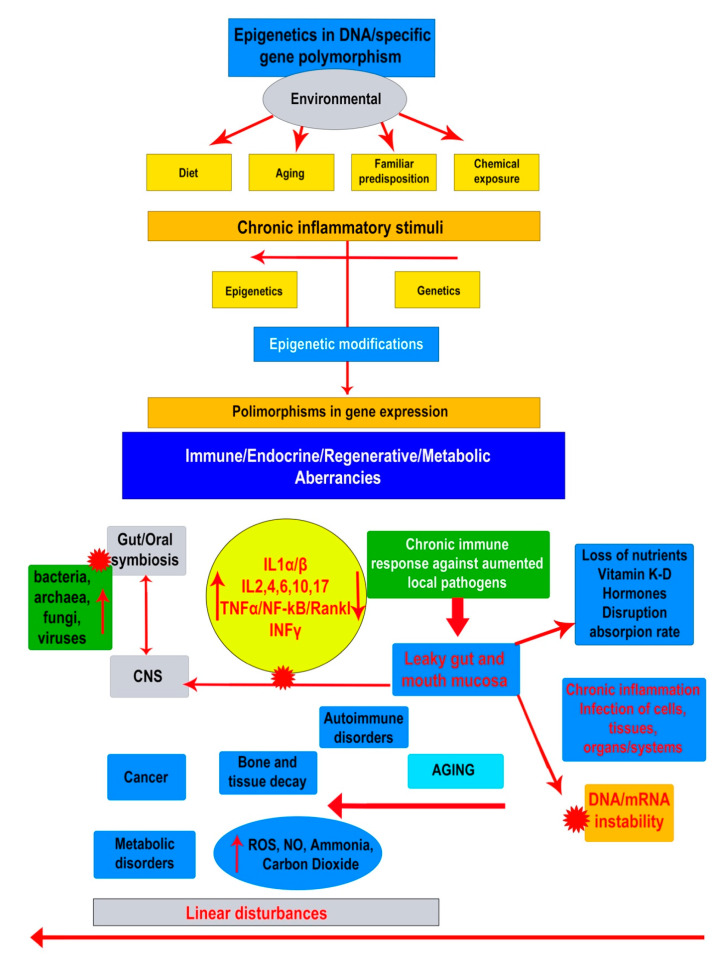
The proposed schematic representation of how the dysbiosis may eventually interfere the whole organism functionality. Environmental risk factors such as life style and food may negatively influence the microbiome raising up the activity of bacteria, fungi and viruses that lead the immune system towards a state of progressive pro-inflammatory activity through the presence of TNF-a, IFNy, Interleukins. Bidirectional signaling between the oral-gastrointestinal tract and central nervous system (CNS) occurs through spinal afferents demonstrate that these two systems showed some similarities in terms of expression of pro-inflammatory cytokines and altered physiological functions and have increased awareness about the epigenome and microbiome, highlighting a plausible link between the gut microbiome and epigenetic modification of the host. This has explained the intensification of various diseases such as immune-mediated, metabolic, and cardiovascular diseases and cancer. Two arrow head indicates e bidirectional communication between two systems. 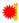 Indicates the high negative impact of agent or group of agents on a different system or another compound, as bacteria, viruses and fungi on oral/gut eubiosis.

Increased interest in oral and gut dysbiosis has given new perspectives in the understanding of etiopathogenesis of important debilitating systemic diseases, such as bone, auto-immune disorders, neuro-degenerative diseases, and cancers. In oral dysbiosis, the presence of CP is a hallmark that is characterized by a steady inflammatory condition promoted by an increased colonization of subgingival biofilms and dental structures by a variety of pathogens both Gram-negative and Gram-positive, anaerobes, and aerobes. The disease has a sturdy progressive evolution based on both the level of host immune responses and the increased accumulation of bacteria and their virulence [23]. By common accordance the most common pathogenic strains involved in CP are *P. gingivalis, T. denticola*, and *T. forsythia*. It is well confirmed that the poly-microbial etiology eventually includes different other microbial species, like those detected in this study, such as *A. actinomycetemcomitans*, *R. dentocariosa*, *C. homini*, *E. corrodens*, *F. alocis*, and *Synergistetes*. Though, the role and relation existing between these pathogens and IL-10 modulation still remain to be fully elucidated, here we have attempted to highlight how they may affect IL-10 genotypes generating specific SNPs (Figure 1).

Nowadays, these polymorphisms are believed to be linked to local environmental influences rather than consequences of genetic or congenitally inherited abnormalities. Therefore, scientists prefer to refer to epigenetics to describe this condition. This position is strongly supported by several lines of evidence that showed how epigenetic modification might eventually exert a great assortment of alterations within the DNA protein assembly mechanism as the main cause of aberrant chromatin transformation that negatively affects gene activity [7]. The DNA epigenetic modifications are characterized by micro damages due to both methylation and histone modifications and are associated with variants in the normal activity of several types of genes directly involved in cytokine and interleukin expression, events that may stay behind an altered IL-10 gene expression [9]. Intriguingly, Larsson’s results showed that DNA methylation assays revealed that this mechanism was seen in blood cells and infected gingival tissues [9]. These outcomes were then confirmed by Moudi and colleagues [11] in their study on IL-10 gene polymorphisms detected in a sample of chronic periodontal patients.

In a different study of IL-10 polymorphisms were seen in correlation with diet and colorectal cancer (CRC). A cohort study, performed by a Danish group, showed how a diet rich in fiber may exert a beneficial effect on CRC through the protection activity of IL-10 among those individuals with genetic susceptibility of IL-10 to CRC [10,11]. Indeed, these data may eventually sustain the evident link between epigenetic mutations typical of the dysbiotic condition in relation to metabolic disorders, and vice-versa. It follows that disorders such as CP, Crohn’s disease, irritable bowel syndrome (IBS), ulcerative colitis, and neoplasia could share the same degenerative pathways and pathological patterns [11,12].

To obtain a better understanding of this standoff, it is important to clarify the intricate nature and function of IL-10. The role of IL-10 in the anti-inflammatory process and its key suppressive role in the pathogenesis of periodontal disease are well known. These roles were confirmed by experiments with IL-10-deficient mice that showed great susceptibility to *P. gingivalis*-induced periodontitis together with high pro-inflammatory phenotypes [24]. In vivo, a great variety of cells may produce IL-10, among them the primary ones are T helper cells (Th), B lymphocytes, dendritic cells (DCs), monocytes, macrophages, and dendritic cells, and even non-immune phenotypes like epithelial cells and keratinocytes. Data from IL-10-deficient mice showed interesting outcomes, as they developed a high susceptibility to other types of suppressed immune mediate diseases, such as chronic enterocolitis and irritable bowel disease, strictly dependent on a complete activation of the polarized Th1 response through cell-derived IL-10 [25,26]. Thus, IL-10 may exert a modulatory effect on MHC class II and costimulatory molecule CD 80-86, on antigen presenting cells (APCs), monocytes, and macrophages, and, in addition, can modulate the expression of pro-inflammatory interleukins, cytokines, and major histocompatibility complexes (MHCs) as IL-1α and β, IL-6, IL-12, IL-18, and TNF-α [26,27].

Within this investigation, we observed the association between specific genetic profiles, oral dysbiosis, bacteremia, and CP. Particular attention was given to genes involved in bone metabolism, such as the VDRs, COLIA1, and the genes in charge of immune responses IL-10, TNF-α, and IL-1α, 1β, and 1RN. Interestingly, there was a significant positive association between the IL-10 ATA/GCC genotypes, the ATA/ACC-ATA/ATA-ACC/ACC genotypes and the relevant incidence of susceptibility to CP was confirmed by the presence of specific bacteria strains like *T. forsythensys* (high aggressive strain), *R. dentocariosa* (medium aggressive strain), *C. hominis* (low aggressive), *P. gingivalis*, *T. forsythensys*, *T. denticola*, *P. micros*, *Synergistetes* (high aggressive strain), and *E. corrodens* (low aggressive strain). However, a similar association was also detected in GCC/GCC genotypes within the third group of 22 patients (IL-10 normal production), with strains that referred to *A. actinomycetemcomitans* (super aggressive strain) and *P. endodontalis* (high aggressive strain) (Table 5).

On the other hand, no significant association was statistically evident between genotype frequencies for the whole IL-10 genotype variation and the group of pro-inflammatory gene type, TNF-α, Bmsl VDR, and COLIA1.

Evidence indicates that periodontal disease with a predominant IL-10 deficit is most common on ATA/ACC haplotypes and this feature tends to be explained by epigenetic disturbances under the effect of both environmental and bacteria activity rather than congenital genetic predisposition. The host immune response has revealed to be a very dynamic mechanism in periodontal diseases in both exacerbation and chronic condition and can be described in terms of patterns of disease, progression under a variety of different immune factors, different competitive pathogens, and dysbiosis that may eventually lead to systemic disorders and bone tissue decay [28]. Loss of proper immune responses, based on the imbalance between pro- and anti-inflammatory factors documented by sequential metabolic assessments, is thought to reflect the cumulative effect of such continuous inflammatory/infection mode and, therefore, may represent the most effective tool in understanding the progressive periodontal disease.

In this study, there was no significant difference in age and sex distributions in the groups (*p* > 0.05). However, the overall prevalence of chronic periodontitis among smokers was estimated at 100%. Currently smokers were found to have significantly higher prevalence (*p* < 0.001) and severity (*p* < 0.001) of periodontitis as compared with past-smokers and non-smokers (data not shown due to the limited number of subjects enrolled in the present pilot study).

In fact, the exacerbation episodes of chronic inflammatory condition, typical of periodontal disease, may be associated with variations in changes of inflammatory patterns, hormones, and related co-existing diseases related to the MetS [2]. Condition like gut diseases, arthritis, and neuro-vegetative decays, show a common clinical condition endorsed by a significant number of mast cells, monocytes/macrophages, and plasma cells when compared to non-infected sites of healthy individuals [29,30,31,32,33,34,35,36,37].

We propose here, in accordance to recent advancements in the periodontal/dysbiosis research, a new model of pathogenesis. This means that periodontal disease, as a chronic condition consequent of long-term abnormal local and systemic bacteria, may trigger the sequelae of genes involved in immune, metabolic, and regenerative responses. These events are, hence, considered as main epigenetic factors that conclude to epigenetic gene polymorphisms. Thus, as shown in our results, gene SNPs detected in periodontal tissues of affected patients related to either IL-10, VDRs, or pro-inflammatory IL-1α, IL-β, IL-RN, and TNF-α, may arise under a common dysbiotic scenario following local and systemic response.

Any statistical incongruity may be attributable to both complexity of the organism, micro-environment (uncoordinated co-existence of multiple factors, like microorganisms and different gene modulation) and quantification analysis issues (potential influence of unknown physiological variables determined by the conflict of anti-inflammatory interleukins versus pro-inflammatory cytokines and interleukins), or to the differences in the size of the cohorts.

## 5. Conclusions

Systemic degenerative diseases are severely influencing the quality of people’s health and life. The clear vision on how we can contribute to the maintenance of body/mental health is by improving the regenerative capacity of our organism, balancing detrimental stimuli. Thus, we hypothesize the utility of understanding the negative impact of dysbiosis, the prominent role of the immune system and endocrine balance as novel therapeutic strategy in degenerative diseases. This clinical approach might be an attractive option to determine a better solution for inflammatory-related bone loss, such in the case of CP. In this study, we demonstrated for the first time, to the best of our knowledge, that the presence of gene variants of the promoter region of the interleukin-10 gene was systematically observed in correlation with gene variants of the IL-1α, IL-1β, and IL-1 RN, gene variants of the VDRs genes (Taql, Apal, Foql, Bsml), and the gene variant of the COLIA1 gene. These observations may eventually be useful to establish the end-point of a risk measurement in developing systemic diseases in CP-affected individuals. Further studies and more genetic information, with a more representative sized sample, are definitively needed to provide an additional understanding of the possible role of interleukin/cytokine gene polymorphisms in a dysbiosis-related chronic condition, aiming to improve diagnosis, evaluate the grade of risk, and validate further effective therapeutic approaches for this disease.

## 6. Patents

The following is a patent resulting from the work reported in this manuscript: 102017000131513 Name: Biomolecular Diagnostic S.r.l. Inventor name: Martelli Francesco. Title: “*Metodo e kit per la diagnosi e la prognosi di parodontite e/o perimplantite*”.

## Figures and Tables

**Table 1:** Value legend.

SNPs	Effects of Polymorphisms Variants
IL-10 (−1082 G > A, −819 C > T, −592 C > A)	-ATA/ATA-ATA/ACC-ACC/ACC: Low production; -ACC/GCC-ATA/GCC: Reduced production; -GCC/GCC: High production.
TNF-α (−308 G > A)	-AA-AG: Predisposition to a higher level of inflammation; -GG: Normal level of inflammation
IL-1α (−889) IL-1β (+3954) IL-1RN (+2018)	No alteration Low alteration/Moderate alteration Severe alteration
TaqI VDR (−1056 T > C)	-tt: Not associated with increased susceptibility of developing periodontal disease and associated with normal serum levels of Vitamin D; -Tt: Greater susceptibility to develop the periodontal disease unrelated to reduced serum levels of Vitamin D; -TT: Greater susceptibility to develop the periodontal disease correlated with reduced serum levels of Vitamin D, with effects on the bone metabolism and immune response
ApaI VDR (+64,978 G > T)	-AA-Aa: Predisposition to osteoporosis; -aa: No predisposition to osteoporosis
BsmI VDR (+63,980 G > A)	-BB-Bb: Predisposition to decrease BMD and to reduce intestinal calcium absorption; -bb: No predisposition to decrease of BMD and to reduce intestinal calcium absorption
FokI VDR (+30,920 T > C)	-FF-Ff: Predisposition to decrease BMD; -ff: No predisposition to decrease BMD
COLIA1 (polymorphism in collagen type-lα) (2046 G > T)	-ss-Ss: Predisposition to osteoporosis; -SS: No predisposition to osteoporosis

**Table 2:** The main periodontal pathogens responsible of oral disease, include Gram-positive and Gram-negative bacteria, facultative, and anaerobic/aerobic bacteria.

Strain Name and Phenotypes	Status of Aggression
*Aggregatibacter actinomycetemcomitans* G−, Ana (fac)	Highly aggressive
*Tannerella forsythensys* G−, Ana	Aggressive
*Porphyromonas gingivalis* G−, Ana	Aggressive
*Treponema denticola* G−, Ana	Aggressive
*Peptostreptococcus micros* (Micromonas) G+, Ana	Aggressive
*Filifactor alocis* G+, Ana	Aggressive
*Synergistetes* G−, Ana	Aggressive
*Porphyromonas endodontalis* G−, Ana	Aggressive
*Fusobacterium nucleatum* ssp. G−, Ana	Medium aggressive
*Campylobacter rectus* G−, Ana (fac)	Medium aggressive
*Prevotella intermedia* G−, Ana	Medium aggressive
*Leptotrichia hofstadii* G−, Ana	Medium aggressive
*Rothia dentocariosa* D+, Aer	Medium aggressive
*Eikenella corrodens* G−, Ana (fac), oral, gut	Low aggressive
*Cardiobacterium hominis* G−, Aer	Low aggressive

“Red” group: *A. actinomycetemcomitans*, *T. forsythensis*, *P. gingivalis*, *T. denticola*, *Peptostreptococcus micros*. The presence of these bacteria is mainly associated with advanced periodontitis (in deep pockets) and perimplantitis. Moreover, also *F. alocis*, *Synergistetes*, and *P. endodontalis* have been considered. *F. alocis* is one of the few bacteria associated to multiple oral pathologies including localized aggressive periodontitis, endodontitis and peri-implantitis. The relative abundance in periodontal pocket of patients with periodontitis may support the hypothesis of including *F. alocis* as a diagnostic marker; *Synergistetes* are opportunistic pathogens, in cases where they have the disease and are part of the red complex of periodontal pathogenic bacteria. *P. endodontalis* can cause periapical lesions with acute symptoms such as pain, swelling, and suppuration; “Orange” group: *F. nucleatum*, *C. rectus*, *P. intermedia*, *L. hofstadii*, *R. dentocariosa*. The presence of these bacteria is mainly associated with the initial or moderate forms of periodontal disease, or in the healing phases; “Green” group: *E. corrodens* and *C. hominis.* The presence of these bacteria is associated with oral health, even if *C. hominis* has been seen in pericardium and heart tissue infection.

**Table 3:** Bacteria used as specific biomarkers for the comparison among the results obtained with massive sequencing on the microbiological samples of the various patient groups. Massive sequencing can identify all the genes recognized by 16S.

Bacteria	Nucleotide Primer Sequence
*Porphyromonas gingivalis* FW *Porphyromonas gingivalis* PROBE *Porphyromonas gingivalis* REV	5′ GCG CTC AAC GGT TCA GCC 3′ 5′ CACTGAACTCAAGCCGGCAGTTTC 3′ 5′ CAC GAA TTC CGC CTG C 3′
*A. actinomycetemcomitans* FW *A. actinomycetemcomitans* PROBE *A. actinomycetemcomitans* REV	5′ GAACCT TACCTACTCTTGACATCC GAA 3′ 5′ AGACTCAGAGATGGGTTTGTGCCTTAGGG 3′ 5′ TGCAGCACCTGTCTCAAAGC 3′
*Tannerella forsythia* FW *Tannerella forsythia* PROBE	5′ACATCGTGCAGGAAGGTGTA 3′ 5′ ACTCGGCAATGACAGGAAGT 3′
*Tannerella forsythia* REV	5′ ACAGGGCGGAGTTGATTACA 3′
*Peptostreptococcus micros* FW *Peptostreptococcus micros* PROBE *Peptostreptococcus micros* REV	5′AGCCATTGAAGACACTTTGGT 3′ 5′AGTGCAGATGTAAAAGTC 3′ 5′ TGCCGAAGTTTCTAGCCAAA 3′
*Fusobacterium nucleatum* FW *Fusobacterium nucleatum* REV	5′ CAACCATTACTTTAACTCTACCATGTTCA 3′ 5′ GTTGACTTTACAGAAGGAGATTATGTAAAAATC 3′
*Prevotella intermedia* FW *Prevotella intermedia* REV	5′ CCTGAGGTCTTCGATGCGTG’ 3′ 5′ TGGGCAAGCATAGACCAAGA 3′
*Campylobacter rectus* FW *Campylobacter rectus* REV	5′ AGCGCAACCCACGTG’3′ 5′ CGCCATTGTAGCACG 3′
*Eikenella corrodens* FW *Eikenella corrodens* PROBE *Eikenella corrodens* REV	5′ ATGTGAAATCCCCGGGCTTA 3′ 5′ CCCTGGGATAACACTGAC 3′ 5′ CTGTTTGCTACCCACGCTTT 3′
*Treponema denticola* FW *Treponema denticola* PROBE *Treponema denticola* REV	5′ ATTTCGACTTTATGCGGGCC 3′ 5′ TCGGCAACAGAAGCATTGTC 3′ 5′ AGGGGATAATTATGGGGCGG 3′

**Table 4:** Bacteria biomarkers, specific from this invention (patent no. 102017000131513).

Bacteria	Sequence (FAM)	Size
*Rothia dentocariosa* FW *Rothia dentocariosa* PROBE *Rothia dentocariosa* REV	5′ ATGATGCAGAACCCCGTACA 3′ 5′ ACACCGAAAAATCGCCCTTC 3′ 5′ TGGGCCTGATGACCTTTTCT 3′	215 pb
*Synergistetes* FW *Synergistetes* PROBE *Synergistetes* REV	5′ TTGAGACTGAGGTGCTGGAG 3′ 5′ TCCCAGTGTAGCGGTGAAAT 3′ 5′ TCTAATCCCGTTCGCTACCC 3′	156 pb
*Filifactor Alocis* FW *Filifactor Alocis* PROBE *Filifactor Alocis* REV	5′ ATACAGTCCGTTTCCACCGT 3′ 5′ ACTCGGCAATGACAGGAAGT 3′ 5′ ACTGATCCTGACCGTTCCTC 3′	172 pb
*Porphyromonas endodontalis* FW *Porphyromonas endodontalis* PROBE *Porphyromonas endodontalis* REV	5′ GCTCAACTGTAGTCTTGCCG 3′ 5′ TGCTAGAGAGGAGACGAGGT 3′ 5′ TGTTTGATCCCCACGCTTTC 3′	168 pb
*Cardiobacterium hominis* FW *Cardiobacterium hominis* PROBE *Cardiobacterium hominis* REV	5′ GAGCCAATCTGAGAAAGCCG 3′ 5′ TACGTTCCCGGGTCTTGTAC 3′ 5′ GCGCCCTCCTAAGTTAAGCT 3′	187 pb
*Leptotrichia buccalis* FW *Leptotrichia buccalis* REV	5 TTGTAAAGGGGATGGCGACT 3′ 5′ GTCCCGCTCCAACAACAATT 3′	248 pb
*Capnocytophaga sputigena* FW *Capnocytophaga sputigena* REV	5′ GCAAGTCGAGGGAGAGGTTA 3′ 5′ GAGCCGTTACCTCTCCAACT 3′	208 pb

**Table 5:** IL-10 polymorphism in the three groups in correlation with other gene expression. IL-10 severe SNPs (ATA/ATA-ATA/ACC-ACC/ACC) included VDRs SNPs, especially seen in Taql, Apal, and Fokl (*p* < 0.05) (red and yellow with Taql TT/Tt, Apal AA/Aa, Foql FF/Ff); IL-10 moderate SNPs (ACC/GCC) included VDRs SNPs seen in VDR Taql, 17 patients TT (red) (*p* < 0.05) and 20 patients Tt (yellow). The SNPs in IL-10 functionally expressed by ATA/ATA-ATA/ACC-ACC/ACC genotypes have no statistically significant relation with COLIA1 gene expression. IL-10 depletion had very low effect on COLIA1 gene expression, genotypes analysis revealed 2 ss (red color), 9 Ss (yellow color), and 19 SS (green color); SNPs in IL-10 functionally expressed by ATA/ATA-ATA/ACC-ACC/ACC genotypes showed a certain degree of correlation with ILs-1 group. Patients with IL-10 depletion showed a substantial affection IL-1gene expression, genotypes analysis showed 10 severe (red color), 17 moderate (yellow color), and seven without alteration (green color). IL-10 polymorphisms (ATA/ATA-ATA/ACC-ACC/ACC) and TNF-α showed a moderate inverted type of correlation. SNPs at IL-10 showed a quite substantial effect on patient’s TNF-α gene expression. Severe IL-10 depletion showed no effect on TNF-α gene activity that was statistically significant (*p* > 0.05). From severe to moderate to normal IL-10 gene expression TNF-α was shown to be always substantially regularly expressed from 65% to 78% to 77% in the GG haplotype, 32%, 23%, and 23% in the AG haplotype and practically 0–3% in AA haplotype (red= severity grade 2; yellow = severity grade 1; green = severity grade 0, normal expression).

Gene Expression	Count (Subjects):	IL-10		
		22	40	34
		GCC/GCC	ATA/GCC	All Severity = 2
	Grade of SNPs and Haplotype	0	1	2
**IL-1** **α/β/RN**	0	32% = 7	35% = 14p	21% = 7p
	1	55% = 12p	35% = 14p	50% = 17p
	2	14% = 3p	30% = 12p	29% = 10p
**TNF-α**	0 GG	77% = 17p	78% = 31p	65% = 22p
	1 AG	23% = 5p	23% = 9p	32% = 11p
	2 AA	0% = 0p	0% = 0p	3% = 1p
**Tagl VDR**	0 tt	5% = 1p	8% = 3p	18% = 6p
	1 Tt	59% = 13p	50% = 20p	41% = 14p
	2 TT	36% = 8p	43% = 17p	41% = 14p
**Apal VDR**	0 aa	14% = 3p	25% = 10p	6% = 2p
	1 Aa	55% = 12p	45% = 18p	71% = 24p
	2 AA	32% = 7p	30% = 12p	24% = 8p
**Bsml VDR**	0 bb	32% = 7p	50% = 20p	44% = 15p
	1Bb	23% = 15p	45% = 18p	50% = 18p
	2 BB	45% = 0p	5% = 2p	6% = 2p
**Fokl VDR**	0 ff	9% = 2p	13% = 5p	3% = 1p
	1 Ff	50% = 11p	55% = 22p	50% = 17p
	2 FF	41% = 9p	33% = 13p	47% = 16p
**COLIA 1**	0 SS	32% = 10p	35% = 15p	21% = 19p
	1 Ss	55% = 9p	35% = 14p	50% = 9p
	2ss	14% = 3p	30% = 1p	29% = 2p
